# Examining the tsetse teneral phenomenon and permissiveness to trypanosome infection

**DOI:** 10.3389/fcimb.2013.00084

**Published:** 2013-11-19

**Authors:** Lee Rafuse Haines

**Affiliations:** Department of Vector Biology, Liverpool School of Tropical MedicineLiverpool, UK

**Keywords:** Glossina, teneral phenomenon, immunity, vector competence, African trypanosomiasis, tsetse symbionts, peritrophic matrix

## Abstract

Tsetse flies are the most important vectors of African trypanosomiasis but, surprisingly, are highly refractory to trypanosome parasite infection. In populations of wild caught flies, it is rare to find mature salivarian and mouthpart parasite infection rates exceeding 1 and 15%, respectively. This inherent refractoriness persists throughout the lifespan of the fly, although extreme starvation and suboptimal environmental conditions can cause a reversion to the susceptible phenotype. The *teneral phenomenon* is a phenotype unique to newly emerged, previously unfed tsetse, and is evidenced by a profound susceptibility to trypanosome infection. This susceptibility persists for only a few days post-emergence and decreases with fly age and bloodmeal acquisition. Researchers investigating trypanosome-tsetse interactions routinely exploit this phenomenon by using young, unfed (teneral) flies to naturally boost trypanosome establishment and maturation rates. A suite of factors may contribute, at least in part, to this unusual parasite permissive phenotype. These include the physical maturity of midgut barriers, the activation of immunoresponsive tissues and their effector molecules, and the role of the microflora within the midgut of the newly emerged fly. However, at present, the molecular mechanisms that underpin the teneral phenomenon still remain unknown. This review will provide a historical overview of the teneral phenomenon and will examine immune-related factors that influence, and may help us better understand, this unusual phenotype.

## The *teneral* phenomenon

**Figure 1:**
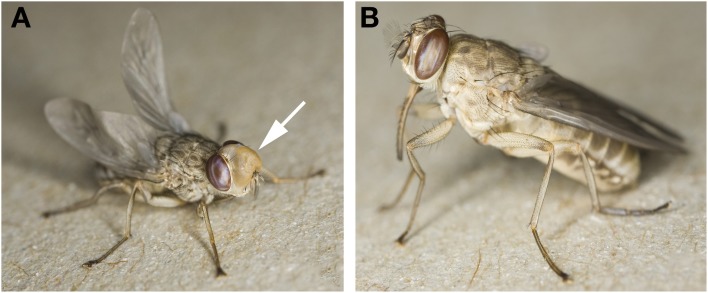
**Newly emerged “teneral” tsetse. (A)** *Glossina morsitans morsitans* <15 min post-emergence from the puparium. The partially inflated ptilinum (marked by arrow) and unfurling wings are hallmarks for the youngest teneral flies. **(B)** *Glossina palpalis palpalis* <2 h post-eclosion. Note the pale body coloration, the ptilinal suture, weak legs, and fully extended wings. Images were used with permission from the photographer, Dr. Ray Wilson. http://www.raywilsonbirdphotography.co.uk/Galleries/Invertebrates/vectors/Tsetse_Fly.html.

The word “*teneral*” is derived from the Latin verb “*tener*,” which means tender, young, and soft. A teneral, newly emerged tsetse fly has not yet ingested a bloodmeal and is quite easily recognized when held. Gently squeezing a teneral fly will often cause the eversible head pouch (ptilinum) to protrude from the fly forehead, which the fly uses to rupture the puparial case (puparium) upon emergence (Figure 1A). Upon emergence, the fly body is of a lighter coloration, has a soft exoskeleton and feels “soapy” to touch. As the fly ages, the ptilinum retracts back into the head to form the ptilinal suture (Figure 1B). After puparial eclosion, a teneral fly is still undergoing stages of physiological maturation. Consequently, there are notable differences in fly behavior, fly physiology, and parasite susceptibility compared to a mature (fed) tsetse (Anderson and Finlayson, 1973). Typically, wild caught teneral *Glossina morsitans morsitans* are inactive during the first 2 days after emergence and rest under shrubs instead of engaging in host and mate-seeking flights (Jackson, 1946). Sexual maturity is also delayed and male *G.m. morsitans* reach sexual maturity 3 days post-emergence, as measured by cessation of accessory gland growth and insemination success (Foster, 1976).

Van Hoof et al. (1937) first observed the “teneral phenomenon” by studying the infection rates of *Trypanosoma brucei gambiense* in *G*. *fuscipes fuscipes*. In this study, it was noted that newly emerged flies that were fed a trypanosome-infected first bloodmeal had higher susceptibility to parasite infection than flies that were fed trypanosomes in subsequent bloodmeals. Wijers (1958) further investigated this “first-day factor” with young *G*. *palpalis palpalis* emergents and demonstrated that flies were more permissive to *T. b. gambiense* midgut infections when fed trypanosomes in the first bloodmeal. This observation confirmed that susceptibility was dependent on the post-eclosion age of the fly upon ingestion of the infectious bloodmeal. Otieno et al. (1983) using *T. b. brucei*, demonstrated that a pronounced teneral phenomenon also existed in newly emerged *G. m. morsitans*. The highest number of salivary gland infections was achieved by feeding infected blood to flies aged <8 h post-emergence (hpe). Distelmans et al. (1982) also confirmed that a different trypanosome species, *T*. *congolense*, had higher infection rates in teneral flies. In this large study consisting of 800 male and female *G. p. palpalis*, only flies aged less than 32 hpe developed mature proboscis infections. This phenotype was evident in both sexes, although to a lesser extent in females (Distelmans et al., 1982; Maudlin et al., 1990). Interestingly, tsetse show susceptibilities that are often produced by specific vector-parasite pairings. For example, age-matched female *G. p. palpalis* are less permissive than *G. m. morsitans* females to trypanosome establishment when they are simultaneously fed the same concentration and strain of *T. b. brucei* (Walshe et al., 2011). Starvation can also impact the vector competence of teneral (unfed) and mature (fed) flies (Kubi et al., 2006). *G. m. morsitans* were subjected to extreme starvation (>72 hpe) prior to infection with either *T. congolense* or *T. b. brucei*. Nutritional stress increased the percentage of flies with mature proboscis and salivary gland infections, respectively. The authors suggested that key immunological responses must be suppressed when starvation depletes fat body lipid reserves. Starvation has been shown to reduce the levels of the fat body-expressed host defense peptides (HDPs), attacin, and cecropin, and this may further contribute to the increased susceptibility to trypanosomes in nutritionally stressed flies (Akoda et al., 2009).

The teneral phenomenon is not caused by trypanosome-specific dynamics. Higher numbers of ingested trypanosomes do not eliminate the age-dependent infection phenomenon as younger teneral flies ingest fewer trypanosomes than older flies (smaller bloodmeals), yet they develop higher parasite infection rates. However, in older teneral flies, parasite load can exacerbate the teneral phenomenon since more trypanosomes are required to produce infectious flies. Walshe et al. (2011) demonstrated that restricting the number of trypanosomes fed to flies (180/fly) produced midgut infection rates of 20% in the young flies (24 hpe) and only 9% in the older flies (48 hpe). Consequently, predicting the infection prevalence in flies based on the total number of trypanosomes ingested can be misleading. Furthermore, Wijers and Willett (1960), working with four human isolates of *T. b. gambiense* in *G. p. palpalis*, reported that infection prevalence was dependent on the number of short-stumpy bloodstream forms ingested by the fly. To determine if the teneral phenomenon was limited to specific parasite life stages, newly emerged flies were given bloodmeals spiked with either *T. b. brucei* bloodstream form or *in vitro* cultured procyclic form trypanosomes (Walshe et al., 2011). Procyclic-fed young tenerals were more susceptible than older tenerals, inferring that the trypanosome lifecycle stage has no effect on the teneral phenomenon. In addition, the monomorphic trypanosome species, *T. congolense* 1/148, was fed to *G. m. morsitans* under the same conditions. Again, the disparity between young and old emergents was evident in both male and female flies (Walshe et al., 2011). No data on teneral susceptibility in *Glossina* sp. infected with *T. vivax* has yet been reported. Although *T. vivax* can undergo cyclical development in *Glossina* (Moloo and Gray, 1989), it is unknown whether the physiological limitations of the immature teneral fly can directly affect trypanosome establishment in mouthpart tissues to facilitate *T. vivax* colonization.

Understanding the teneral phenomenon and what factors influence it is important not only for lab-based studies but also for tsetse fieldwork. Biological factors, such as the presence and spatial distribution of teneral and non-teneral tsetse, are used to characterize potential hot spots of trypanosomiasis transmission (Grébaut et al., 2009). Monitoring teneral flies is also crucial to gauge the efficacy and specificity of different vector control programmes (Tchouomene-Labou et al., 2013).

## Midgut immunity

**Figure 2:**
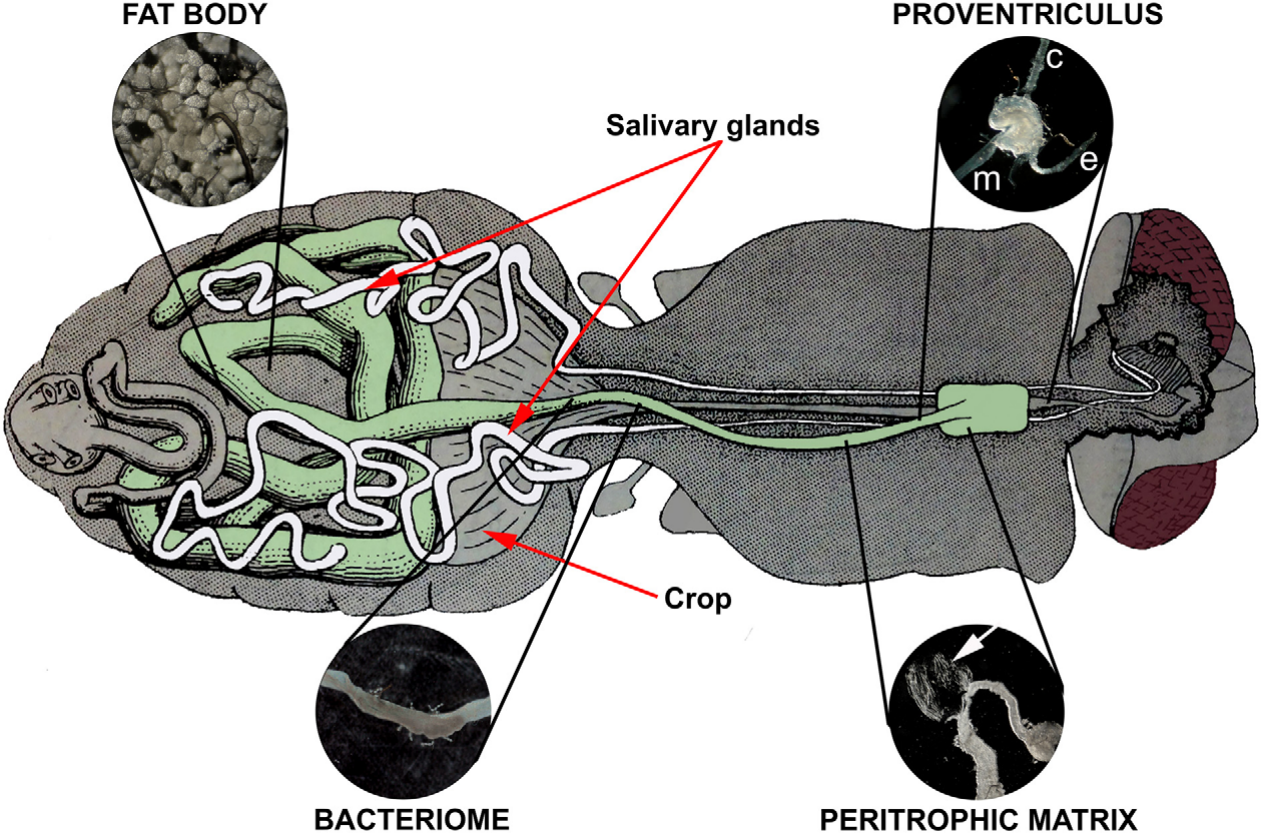
**Internal anatomy of the tsetse fly showing the tissues associated with fly refractoriness to parasite infection: proventriculus, peritrophic matrix, bacteriome, and fat body.** Midgut-associated tissues (proventriculus, anterior midgut, bacteriome, mid and hindgut) are shaded in green. The fat body is dispersed throughout the abdomen; the inset depicts a characteristic grapelike cluster. The proventriculus encloses the junction between the esophagus (e), crop (c), and anterior midgut (m). A section of the peritrophic matrix is shown as a transparent sheath (arrow) extruding from a nicked anterior midgut (just posterior to the proventriculus). The bacteriome houses the primary endosymbiont *Wigglesworthia glossinidia* and is observed as a darker, flattened ring surrounding a section of the teneral midgut. The tsetse alimentary canal drawing was adapted from Roubaud (1909; Figure 84: Organization digestive de la *Gl. palpalis*).

Tsetse possess a Type II peritrophic matrix (PM), which is continuously secreted by the proventriculus (PV; cardia) in the anterior midgut (Figure 2) (Moloo et al., 1970; Tellam et al., 1999). The PM forms a long, sheath-like, multilaminate structure that encases each ingested bloodmeal and protects the midgut epithelium from digestive abrasion. This acellular sleeve also forms a physical barrier that bloodmeal-derived pathogens must breach to gain access to the midgut epithelium (Lehane, 1997; Hegedus et al., 2009). Between the PM and the midgut epithelium is a region called the ectoperitrophic space. Trypanosomes must penetrate through the PM to gain access to the ectoperitrophic space (Freeman, 1973; Ellis and Evans, 1977) where they multiply and continue to differentiate (Gibson and Bailey, 2003). In three species of tsetse, Wigglesworth (1929) observed that PMs isolated from teneral flies were “ragged and discontinuous” and failed to extend along the entirety of the midgut. Lehane and Msangi (1991) measured the synthesis of the PM in newly emerged *G. m. morsitans* and determined that the PM requires 84 hpe to fully extend along length of the midgut. The increasing length of the PM correlates to an increase in fly refractoriness to trypanosome establishment (Walshe et al., 2011). Weiss et al. (2013) have recently suggested that the PM can also indirectly influence trypanosome establishment. The authors described an elegant model whereby the partially formed teneral PM gives ingested trypanosomes unrestricted access to the immunosensory midgut epithelium. The premature contact between the epithelia and trypanosome antigens induced a tempered humoral and epithelial immune response that ultimately failed to protect the fly from trypanosome establishment.

The tsetse EP protein (Chandra et al., 2004) is primarily localized to the fly PM and the midgut lumen. It is associated with tsetse immunity and is upregulated in response to bacterial challenge (Haines et al., 2005) and trypanosome infection (Haines, 2009). When tsetse EP protein levels were reduced using RNA interference (RNAi), trypanosome establishment in the fly midgut significantly increased (Haines et al., 2010), thus implying a role in vector competence. Expression of tsetse EP protein is lower in both teneral and trypanosome-susceptible aposymbiotic flies compared to mature, refractory fly midguts, which have a high expression level (Weiss et al., 2013). These data, combined with the compromised barrier of the teneral PM, further support the theory that a teneral midgut is both physically and molecularly impaired and cannot adequately protect the fly against bloodmeal-acquired pathogens.

Peptidoglycan Recognition Protein (PGRP-LB) is an essential tsetse protein expressed in the bacteriome (a specialized organ in the midgut that harbors the obligate symbiont, *Wigglesworthia glossinidia*) (Figure 2) and also the milk glands of female flies. PGRP-LB regulates host immunity by inhibiting the production of antibacterial HDPs. *W*. *glossinidia* appears to control the expression of PGRP-LB in order to attain immune tolerance within the fly. In fact, when PGRP-LB is downregulated using RNAi, antimicrobial peptide synthesis increases, and symbiont numbers consequently decrease (Wang et al., 2009). Curiously, these PGRP-LB knockdown flies become more susceptible to trypanosome infections, despite higher concentrations of HDPs in the midgut. Newly emerged flies cannot express PGRP-LB until they receive a bloodmeal and only low amounts of PGRP-LB are passively provided in the larval meal. Additionally, recombinant PGRP-LB is trypanocidal to bloodstream and procyclic form trypanosomes (Wang and Aksoy, 2012). Thus, teneral midguts contain lower levels of the trypanotoxic PGRP-LB than fed flies, which could explain the lack of refractoriness to infection in young flies.

## Proventricular immunity

The PV is a mushroom shaped structure located at the anterior end of the foregut (Figure 2). It secretes the PM that surrounds the incoming bloodmeal and provides the first line of defense against blood-borne pathogens. Trypanosomes successfully establishing in the fly midgut must penetrate through the PM in the anterior midgut in order to invade the PV (Ellis and Evans, 1977), where they further differentiate into elongated proventricular trypomastigotes (Sharma et al., 2009). This migration constitutes another bottleneck to transmission as only 40% of all *T. b. brucei* midgut infections overcome the proventricular barrier to develop into mature salivary gland infections (Peacock et al., 2012). The PV also expresses immune effector molecules, such as attacin, which can be lethal to trypanosomes. Interestingly, Hao et al. (2003) reported that although the PV transcriptionally regulated attacin and defensin upon bacterial challenge, trypanosome infection did not elicit a response.

Reactive oxygen species are also part of the tsetse innate immune response as they are thought to promote trypanosome death in the midgut. Adding antioxidants to the tsetse bloodmeal eliminates the teneral phenomenon as flies become completely susceptible to trypanosome infection (MacLeod et al., 2007). Nitric oxide synthase activity can be directly correlated to the generation of the reactive oxygen species, nitric oxide, which is known to be trypanocidal (Bogdan, 2001). As expected, the PVs isolated from newly emerged flies contained significantly lower nitric oxide synthase activity compared to fed control flies (Hao et al., 2003). It is questionable whether the PV plays a role in the teneral phenomenon, as the few identified effector molecules secreted and/or regulated by the PV tend to respond to well-established midgut infections rather than to trypanosomes arriving in an infectious first bloodmeal.

## Fat body immunity

The tsetse fat body is loosely distributed throughout the hemocoel and is functionally related to both adipose tissue and the mammalian liver (Figure 2). This multifunctional tissue provides an energy reserve in the form of stored lipids and is a key controller of the innate immune system. It is essential for teneral flies to acquire several bloodmeals to build up enough energy (lipid stores) to develop protective immunity against pathogens. Early research has shown that the fat body contents from teneral *G. palpalis* (Mellanby, 1936) and *G. morsitans* (Jackson, 1946) contain less than 1.0 mg of fat compared to greater than 2.0 mg of fat in fed flies. HDPs are small, evolutionary conserved antimicrobial and antiparasitic molecules that are produced by a diverse number of organisms, including insects (Mor, 2009). Four HDPs have thus far been characterized in tsetse: attacin, defensin, diptericin, and cecropin (Hao et al., 2001; Boulanger et al., 2002), but with the pending release of the tsetse genome, more are likely to be found. Two HDPs, attacin and defensin, are upregulated only after trypanosomes establish in the mature fly midgut (Hao et al., 2001). However, in teneral flies HDP expression does not increase even 5 days after trypanosomes are imbibed (Akoda et al., 2009). Tsetse attacin provides resistance to trypanosome infection and also constrains trypanosome levels in flies if they do become infected (Hao et al., 2001). Nayduch and Aksoy (2007) observed elevated attacin levels in the fat body, midgut, and PV of different fly species known to be naturally less permissive to trypanosome infection, thus directly implicating attacin as a key molecule used to inhibit trypanosome establishment. To confirm the role in tsetse refractoriness, attacin knockdown by RNAi increased both midgut and salivary gland infections. This was the first time an HDP was directly linked to parasite transmission (Hu and Aksoy, 2006). The observed delay in HDP activation by ingested trypanosomes may be exacerbated in teneral flies as a result of an immature immune system and minimal lipid resources present in the fat body. It is also possible that other immune effector molecules are underexpressed or inactive in teneral flies because of similar nutritional restrictions.

## Microbiota-induced immunity

Welburn and Maudlin (1992) proposed that teneral susceptibility to trypanosomes is a maternally inherited trait because (1) the teneral phenomenon disappears in non-teneral flies and (2) it is not dependent on bloodmeal or parasite-derived factors. Tsetse flies are viviparous insects; they nurture their larval instar stages *in utero* on secretions from modified female accessory reproductive glands termed milk glands (Ma and Denlinger, 1974; Attardo et al., 2008). These secretions predominantly consist of lipids and proteins in equal proportion and also contain symbiotic bacteria (Leak, 1998). Teneral flies emerge with remnants of the larval meal still in their midgut. The rapid digestion of the larval meal post-eclosion was shown to co-correlate with age-specific refractoriness to trypanosome infection (Walshe et al., 2011). The larval meal may be a reservoir of lectin inhibitory molecules derived from either the tsetse milk or the symbionts. Teneral flies have very low levels of a secreted trypanosome-killing lectin in their midguts compared to mature flies (Welburn et al., 1989). Several tsetse lectin-binding proteins have since been described (reviewed in Leak, 1998) that are thought to agglutinate trypanosomes in the midgut and inhibit parasite establishment (Welburn et al., 1994). One such inhibitor, D+ glucosamine, is produced by chitin metabolism and prevents tsetse midgut lectin from agglutinating trypanosomes (Ibrahim et al., 1984; Maudlin and Welburn, 1987). When teneral *G. m. morsitans* were co-fed D+ glucosamine with an infective bloodmeal, the fly midgut infection rates tripled, but the ratio of mature infections remained unchanged (Mihok et al., 1992).

The tsetse midgut is also host to the maternally inherited, midgut-associated bacterial symbionts *Sodalis glossinidius* (Welburn et al., 1987; Dale and Maudlin, 1999) and *Wigglesworthia glossinidia* (Aksoy, 1995), which are known to modulate tsetse fecundity and longevity, provide nutritional supplementation (vitamin biosynthesis), and influence vector competence (Dale and Welburn, 2001; Pais et al., 2008; Weiss and Aksoy, 2011). The ratio of *S. glossinidius* to *W. glossinidia* densities significantly increases as teneral and adult flies age (Soumana et al., 2013) until populations reach homeostasis in the mature fly (Snyder et al., 2010). The impact *W. glossinidia* may have on teneral susceptibility via PGRP-LB expression has been previously described in the section on midgut immunity. Since teneral flies harbor fewer *S. glossinidius* compared to adult flies, it is unclear how this limited population could increase susceptibility to trypanosomes unless a threshold number of *S. glossinidius* can stimulate (prime) an antibacterial immune response that is hostile to trypanosomes. Interestingly, when tsetse flies were injected with *E. coli* prior to feeding a trypanosome-infected bloodmeal, they were less susceptible to trypanosome infection (Hao et al., 2001). Therefore, priming the teneral tsetse immune system could increase fly refractoriness to parasite establishment as the teneral fly ages and symbiont populations simultaneously expand. There are also strong indications that *S. glossinidius* increases non-teneral fly susceptibility to parasite infection (Reinhardt et al., 1972; Hecker and Moloo, 1981; Maudlin and Dukes, 1985; Maudlin and Ellis, 1985). In fact, data collected from a large field survey in Cameroon and Angola showed a 3-fold increase in vectorial capacity when tsetse harbored *S. glossinidius* (Farikou et al., 2010). Unfortunately, it is impossible to determine if these fly populations were infected with trypanosomes as tenerals.

## Conclusions

The age-dependent susceptibility of young, unfed teneral flies to trypanosome infection is unusual as most insect vectors become more permissive to pathogens with age (immunosenescence). The teneral phenomenon is influenced by several factors including nutritional stress, bloodmeal parasitaemia, and the density of the microbial population in the fly midgut upon eclosion. However, the specific mechanism(s) underpinning this phenomenon have not yet been confirmed. It is important to understand the limitations of this phenotype, particularly when laboratory experiments assess or compare the vectorial capacity of tsetse. And likewise, in the field, the identification of teneral flies with increased vectorial capacity should be factored into vector control implementation and surveillance programmes.

### Conflict of interest statement

The authors declare that the research was conducted in the absence of any commercial or financial relationships that could be construed as a potential conflict of interest.
